# Enabling ankle-brachial index prediction from doppler sounds using deep learning

**DOI:** 10.1038/s44325-026-00116-7

**Published:** 2026-04-09

**Authors:** Adrit Rao, Kevin Battenfield, Arash Fereydooni, Akshay Chaudhari, Oliver Aalami

**Affiliations:** 1 Stanford Mussallem Center for Biodesign, Stanford, CA USA; 2 Vascular Surgery Medical Group, San Leandro, CA USA; 3 Division of Vascular Surgery, Department of Surgery, Stanford University School of Medicine, Palo Alto, CA USA; 4 Department of Radiology / Integrative Biomedical Imaging Informatics, Stanford University School of Medicine, Palo Alto, CA USA

**Keywords:** Health care, Peripheral vascular disease

## Abstract

The ability to perform accurate point-of-care assessment of limb perfusion is critical for safe clinical decision-making. A formal ankle-brachial index (ABI) is typically required prior to supervised exercise therapy (SET) for peripheral arterial disease (PAD) or compression therapy for venous stasis ulcers. However, ABI measurements cannot be reported in patients with calcified, non-compressible tibial vessels. In this study, we introduce AutoABI, a deep learning algorithm that classifies ABI categories directly from circulatory Doppler sounds to improve the accessibility of point-of-care ABI assessment. AutoABI was trained and tested on a limited size dataset of 791 recordings from 198 patients and predicts ABI categories of <0.5, 0.5–0.7, 0.7–0.9, and >0.9. The algorithm achieved strong discriminatory performance with average areas under the receiver operating characteristic curve (AUCs) of 0.95, 0.96, 0.94, and 0.97 for the respective ABI ranges. Additional testing demonstrated the ability to predict ABI categories in patients with non-compressible arteries, offering a promising solution for more accessible and reliable PAD assessments.

## Introduction

Peripheral arterial disease (PAD) is estimated to affect over 200 million people worldwide and is one of the leading causes of limb loss causing life-long disability^1,2^. Early diagnosis is crucial in enabling timely treatment and preventing adverse clinical outcomes. Current methods of clinical evaluation at the point-of-care consist of skin inspection (loss of hair growth, acrocyanosis, and ulcers), pulse examination with palpation of pedal arteries, and continuous wave doppler evaluation of pedal arteries. The ankle-brachial index (ABI) measurement as an objective PAD screening evaluation was first described by Winsor in 1950^3^. This technique requires the measurement of bilateral brachial and tibial pressures with blood pressure cuffs using a doppler to assess arterial flow to derive an ABI. Unfortunately, the ABI continues to be underutilized at the point of care due to time constraints (estimated at 15 minutes) and lack of reimbursement^4,5^. Formal evaluation in a vascular ultrasound laboratory (such as in our study) requires a separate clinical encounter which can lead to delays in diagnosis and treatment. In addition, ABI studies are challenged by the inability to assess patients with non-compressible tibial vessels, such as those with diabetes mellitus and end-stage renal disease^6^. In a Finnish study, as high as 8% of patients being referred for vascular evaluation had elevated ABIs from calcified tibial arteries, and of these 62% had PAD^7^.

Deep learning enables computer algorithms to extract latent relationships in complex datasets and has proven to have multiple useful clinical applications, with a particular focus on medical imaging^8,9^. Other applications, such as the classification of heart auscultation sounds to classify murmurs, have leveraged deep learning-based sound analysis techniques within the cardiovascular field^10^. Another example of this is the classification of breath and respiratory sounds to diagnose COVID-19^11^. The hand-held continuous wave doppler is a ubiquitous tool used at the point-of-care enabling practitioners to subjectively assess waveform phasicity, another metric utilized for assessing PAD severity. Waveforms are classified into triphasic (normal), biphasic (mild insufficiency), and monophasic (severe insufficiency) categories. Standard dopplers only provide audio output making the interpretation of sounds quite subjective. The ABI remains one of the most accurate ways to assess limb perfusion, however, its limitations as described make it underutilized. Through the use of a deep learning-based approach, we enable the ability to derive clinically relevant ABI ranges directly from doppler sounds, negating the need for the time-consuming, cumbersome, tibial compression-based measurement. We additionally test the ability to predict ABI ranges in non-compressible tibial vessels. With such a tool, we can provide more confidence in deriving the ABI at the point-of-care with increased efficiency and accessibility in evaluation.

## Results

### ABI clinical study outcome

A total of 791 four-second CW Doppler audio recordings were collected from 198 patients in our prospective IRB-approved clinical study. These patients were referred for formal ankle-brachial index (ABI) evaluations at our IAC-accredited vascular laboratory, encompassing individuals with a range of vascular pathologies including peripheral arterial disease, diabetes mellitus, and suspected non-compressible arteries. To prepare the data for deep learning, we applied our custom waveform phase detection algorithm that segmented the audio recordings into discrete one-second windows containing diagnostically relevant signal activity. This preprocessing step yielded a total of 2,082 one-second audio clips, each of which was transformed into a time–frequency spectrogram and labeled according to ABI class. The dataset included 295 one-second clips labeled as ABI < 0.5, 500 as 0.5–0.7, 374 as 0.7–0.9, and 913 as >0.9.

### Evaluation of model performance

To assess the efficacy of deep learning in classifying ABI ranges directly from Doppler waveform data, we experimented with two CNN backbones (ResNet-10 and ResNet-18) within our signal processing-based AutoABI architecture. The models were tasked with classifying inputs into four clinically meaningful ABI categories: <0.5, 0.5–0.7, 0.7–0.9, and >0.9. Evaluation was performed using 5-fold cross-validation, with stratified test sets comprising 20% of the data in each fold. Performance was quantified using class-wise precision, recall, F1-score, and area under the receiver operating characteristic curve (AUROC), summarized in Table 1 (ResNet-10) and Table 2 (ResNet-18).

**Table 1:** ResNet-10 backbone statistical metric performance with 95% confidence intervals

ABI Class	Precision ± 95%CI	Recall ± 95%CI	F1-score ± 95%CI	AUC ± 95%CI
<0.5	0.80 ± 0.11	0.80 ± 0.09	0.80 ± 0.10	0.97 ± 0.01
0.5–0.7	0.86 ± 0.05	0.85 ± 0.03	0.86 ± 0.03	0.97 ± 0.01
0.7–0.9	0.76 ± 0.02	0.82 ± 0.06	0.79 ± 0.03	0.95 ± 0.02
>0.9	0.92 ± 0.02	0.91 ± 0.03	0.92 ± 0.02	0.98 ± 0.01

**Table 2:** ResNet-18 backbone statistical metric performance with 95% confidence intervals

ABI Class	Precision ± 95%CI	Recall ± 95%CI	F1-score ± 95%CI	AUC ± 95%CI
<0.5	0.82 ± 0.06	0.87 ± 0.08	0.84 ± 0.05	0.95 ± 0.03
0.5–0.7	0.89 ± 0.04	0.86 ± 0.04	0.87 ± 0.02	0.96 ± 0.01
0.7–0.9	0.77 ± 0.09	0.81 ± 0.08	0.78 ± 0.05	0.94 ± 0.03
>0.9	0.93 ± 0.03	0.91 ± 0.04	0.92 ± 0.04	0.97 ± 0.02

The ResNet-10 model demonstrated strong overall classification performance across ABI categories. The model achieved F1-scores of 0.80 ± 0.10, 0.86 ± 0.03, 0.79 ± 0.03, and 0.92 ± 0.02 for the <0.5, 0.5–0.7, 0.7–0.9, and >0.9 categories, respectively. Corresponding AUROC values were 0.97 ± 0.01, 0.97 ± 0.01, 0.95 ± 0.02, and 0.98 ± 0.01. The highest performance was observed in the >0.9 category, with a precision of 0.92 ± 0.02, recall of 0.91 ± 0.03, F1-score of 0.92 ± 0.02, and AUROC of 0.98 ± 0.01, indicating excellent discrimination of normal vascular physiology. Performance was lowest in the intermediate 0.7–0.9 category, which exhibited a precision of 0.76 ± 0.02, recall of 0.82 ± 0.06, F1-score of 0.79 ± 0.03, and AUROC of 0.95 ± 0.02. This relative reduction is expected, as this ABI range represents a transitional zone between mild disease and normal perfusion, where Doppler waveform characteristics and phasicity patterns show greater overlap and clinical ambiguity.

The ResNet-18 model yielded consistently strong performance and demonstrated slight improvements in stability and discrimination across ABI categories. The model achieved F1-scores of 0.84 ± 0.05, 0.87 ± 0.02, 0.78 ± 0.05, and 0.92 ± 0.04 for the <0.5, 0.5–0.7, 0.7–0.9, and >0.9 categories, respectively. Corresponding AUROC values were 0.95 ± 0.03, 0.96 ± 0.01, 0.94 ± 0.03, and 0.97 ± 0.02. The highest class-wise performance was again observed in the >0.9 category, with a precision of 0.93 ± 0.03, recall of 0.91 ± 0.04, F1-score of 0.92 ± 0.04, and AUROC of 0.97 ± 0.02. The 0.7–0.9 category remained the most challenging, with a precision of 0.77 ± 0.09, recall of 0.81 ± 0.08, F1-score of 0.78 ± 0.05, and AUROC of 0.94 ± 0.03, reflecting persistent overlap in Doppler waveform morphology at clinically ambiguous ABI thresholds.

**Fig. 1:**
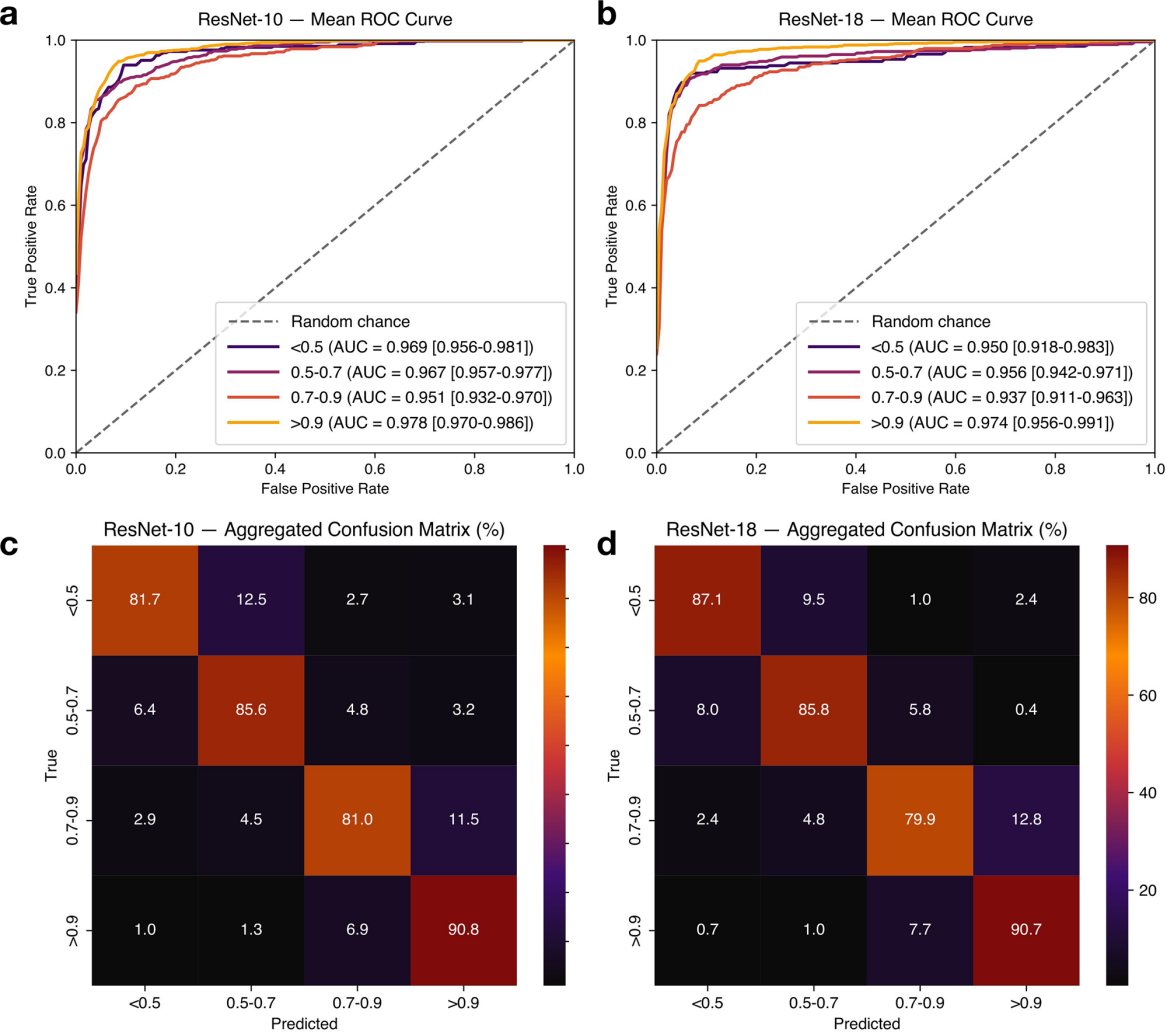
Visualizations of model performance. **a** ResNet-10 Backbone Mean ROC Graph, **b** ResNet-18 Backbone Mean ROC Graph, **c** ResNet-10 Aggregated Confusion Matrix, **d** ResNet-18 Aggregated Confusion Matrix.

The ROC curves for both models are shown in Fig. 1a (ResNet-10) and 1b (ResNet-18), where each curve represents the mean ROC per ABI class. Figure 1c and d show the aggregated confusion matrices for both models, revealing high class-specific agreement, particularly for the 0.5–0.7 and >0.9 categories, and most misclassifications occurring between adjacent ABI ranges, especially between 0.7–0.9 and >0.9.

**Fig. 2:**
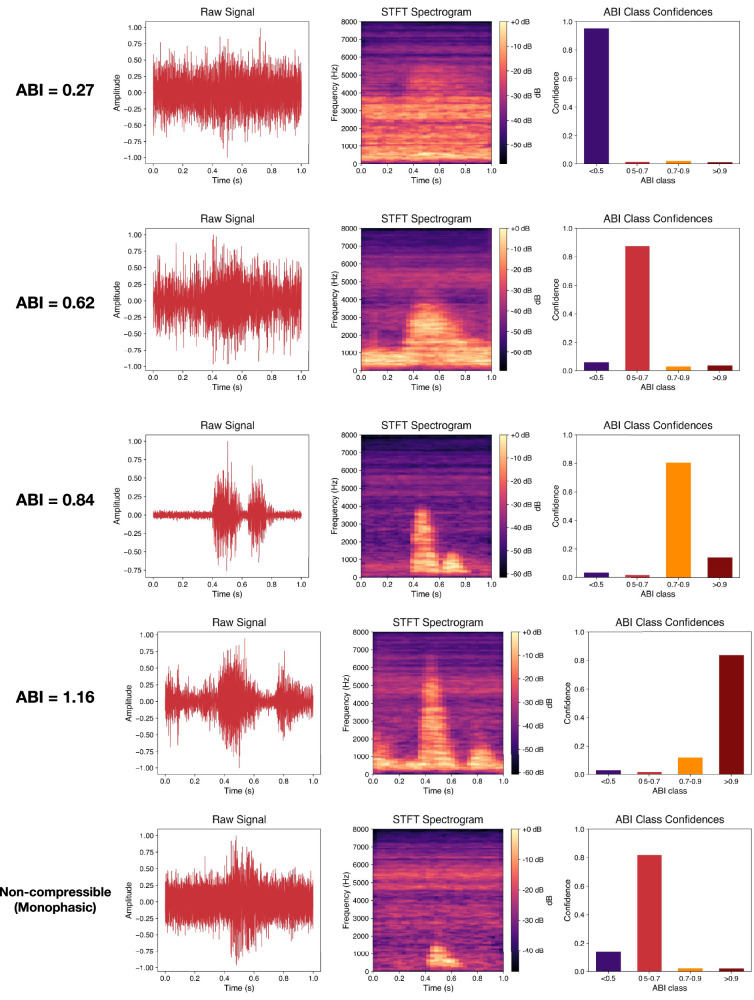
Sample inference per ABI range with non-compressible (Monophasic) sample. Plots consist of raw signal wave, generated spectrogram phase plot, and histogram of predictions with confidence outputted by model.

To explore the model’s interpretability and output behavior, we visualized sample-level predictions across ABI classes. Figure 2 presents five representative cases for both compressible and non-compressible tibial arteries. Each row shows the raw audio waveform, the corresponding STFT spectrogram input, and the output confidence histogram across ABI classes.

### Validation on non-compressible tibial arteries

Given the clinical relevance of evaluating patients with medial arterial calcification and incompressible vessels, we further validated model performance on a subset of ten non-compressible tibial artery recordings collected from diabetic patients. In such patients, traditional ABI measurements based on pressure ratios are often artificially elevated due to arterial stiffening, rendering them diagnostically inaccurate.

We used waveform phasicity as a proxy for hemodynamic status and ground truth reference. Waveform phasicity is a well-established surrogate marker of peripheral perfusion, especially in scenarios where compressibility is impaired. It has been shown to correlate with ABI values and lower extremity arterial disease severity. Specifically, monophasic waveforms are strongly associated with significant arterial obstruction and ABI values < 0.5, while triphasic waveforms indicate normal perfusion and ABI > 0.9, with biphasic signals falling in intermediate ranges^12^. Inter-rater agreement for waveform phasicity classification was assessed using ABI testing waveform recordings for all 10 non-compressible tibial artery cases. Cohen’s kappa was 0.85 (95% CI: 0.51–1.00), indicating substantial to excellent agreement between the two vascular surgeons. Agreement was complete for both monophasic waveforms (100%, 4/4) and triphasic waveforms (100%, 4/4), with 1 discordant case among the 2 biphasic recordings where one rater classified the waveform as triphasic based on ABI machine waveform morphology. This single discordant case was resolved through consensus review of the ABI testing waveform tracing and classified as biphasic based on subtle dampening of the reverse flow component. The correlation between ABIs and expected waveforms are shown in Table 3.

**Table 3:** ABI range and expected waveform phasicity

ABI Range	Expected Waveform
> 0.9	Triphasic
0.7–0.9	Triphasic / Biphasic (transitional)
0.5–0.7	Biphasic / Monophasic (transitional)
< 0.5	Monophasic

As shown in Table 4, all ten predictions made by the model were fully concordant with the expected ABI class derived from waveform phasicity. Monophasic signals were consistently classified as <0.5, biphasic signals as 0.5–0.7, and triphasic signals as either 0.7–0.9 or >0.9 based on waveform robustness. This high concordance supports the models’ potential to accurately predict ABI ranges in non-compressible tibial vessels. However, a separate validation on a significantly larger cohort of patients with non-compressible vessels is needed to fully establish the model’s generalizability.

**Table 4:** 10 diabetic patients tested with non-compressible tibial vessels. Deep learning ABI prediction and ground truth phasicity

Non-compressible Recording Sample	ABI Prediction	Ground Truth Phasicity	Performance
1	0.5–0.7	Monophasic	Concordant
2	0.5–0.7	Biphasic	Concordant
3	0.7–0.9	Triphasic	Concordant
4	> 0.9	Triphasic	Concordant
5	0.5–0.7	Biphasic	Concordant
6	< 0.5	Monophasic	Concordant
7	> 0.9	Triphasic	Concordant
8	< 0.5	Monophasic	Concordant
9	0.5–0.7	Biphasic	Concordant
10	0.7–0.9	Triphasic	Concordant

All tests were concordant.

### Clinical case evaluation

**Fig. 3:**
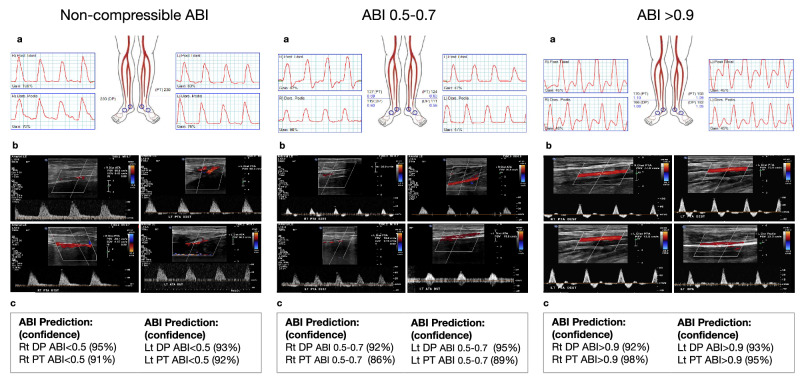
Real-world clinical testing samples. Panel (**a**) shows continuous doppler waveforms from ABI testing machines, Panel (**b**) shows spectral doppler ultrasound waveforms, Panel (**c**) lists the ABI predictions outputted by the algorithm. Patient details - (Left): 75 year old female with diabetes mellitus who has had bilateral toe amputations, (Middle): 92 year old male presents with bilateral lower extremity claudication symptoms with walking approximately one block, resolved with five minutes of rest. Approximate four year history of symptoms and worse in the past twelve months. Risk Factors include: hypertension and hyperlipidemia, (Right): Patient presents for screening exam and does not report claudication symptoms.

To evaluate the model’s real-world applicability, we reviewed predictions in three patients from the test dataset with compressible and non-compressible tibial vessels. Figure 3 presents multimodal outputs for each patient, including continuous Doppler waveform tracings (Panel A), spectral Doppler ultrasound images (Panel B), and model-predicted ABI class with confidence (Panel C). The first patient, with monophasic waveforms and calcified vessels, was correctly classified in the <0.5 range. The second patient showed biphasic flow and was classified in the 0.5–0.7 range. The third patient exhibited triphasic signals and was accurately predicted to have an ABI > 0.9. These cases highlight the potential for automated classification to support clinical interpretation and augment diagnostic confidence in vascular labs.

## Discussion

We have developed a deep learning algorithm to accurately predict ABI ranges from circulatory audio recordings of continuous wave doppler recordings. This end-to-end computational system was developed to make the objective evaluation of limb perfusion more accessible at the point-of-care and across the care continuum, leveraging the ubiquitous hand-held doppler. The current clinical workflow requires patients to have a formal ABI study in a vascular laboratory, for example, before the initiation of compression therapy for edema or being started on a supervised walking program for PAD. With an objective point-of-care ABI prediction tool, earlier diagnosis and treatment can be initiated before the formal ABI study is completed for confirmation.

While automated ABI measurement systems based on oscillometric blood pressure devices, such as the MESI ABPI MD and Form 5 (Fukuda), are commercially available and provide rapid ABI measurements, our proposed deep learning-based approach offers several distinct advantages that warrant consideration for specific clinical contexts. A detailed comparative analysis of AutoABI versus commercial automated systems is provided in Supplementary Table 1, with key differences in measurement time, operator burden, equipment requirements, and failure modes across different patient populations. Traditional automated systems still require multiple blood pressure cuffs, standardized positioning, and cannot overcome the fundamental limitation of calcified, non-compressible vessels that yield falsely elevated or unmeasurable ABI values. In contrast, our doppler sound-based approach leverages the ubiquitous handheld continuous wave doppler already present in most clinical settings, potentially reducing equipment costs and setup complexity. The primary advantage of our method lies in its ability to provide ABI range predictions even in patients with non-compressible tibial vessels, where traditional pressure-based measurements fail entirely and alternative measures such as toe pressures, Toe-Brachial Indices (TBIs), or Plantar Acceleration Times (PATs) are typically required to assess arterial insufficiency severity^13^. Our preliminary analysis of 10 calcified non-compressible tibial vessels showed full concordance with “expected” waveforms, though future studies with TBI and PAT concordance are warranted. However, our approach currently provides ABI ranges rather than precise values, requires validation across different doppler devices and clinical environments, and may be more susceptible to operator technique variations and ambient noise compared to standardized oscillometric devices. As detailed in Supplementary Table 1, the complementary nature of these approaches suggests that doppler-based ABI prediction could serve as a valuable adjunct to traditional methods, particularly in resource-limited settings, emergency departments, or when evaluating patients with calcified vessels where commercial systems would fail or provide misleading results. The complementary nature of these approaches suggests that doppler-based ABI prediction could serve as a valuable adjunct to traditional methods, particularly in resource-limited settings or when evaluating patients with calcified vessels.

In prior studies, machine learning has been implemented to help with interpretation of ultrasound studies. The level of arterial occlusive disease was predicted with an accuracy between 88% and 90% from lower extremity arterial duplex studies using a hierarchical neural network (HNN)^14^. Carotid stenosis prediction from carotid ultrasound studies using velocities achieved an accuracy of 99%^14^. We have not identified prior work which uses continuous wave doppler sound characteristics to predict ABI measurements with deep learning.

The integration of this algorithm onto a mobile device would allow for an inexpensive, objective, point-of-care assessment of limb perfusion through the prediction of ABI ranges. Extensive validation is still required at different clinical sites as well as using various continuous wave doppler machines, as deep learning algorithms are at risk for overfitting training populations and technical bias^15^. Successful implementation of such a system in the real world would also require continuous monitoring and maintenance to account for prediction drift. This algorithm was developed from data of real-world patients being referred to a vascular lab for peripheral vascular disease evaluation, including patients with diabetes mellitus.Validation in other clinical settings such as outpatient clinics and emergency rooms will be necessary.

This study has multiple limitations. The recording of doppler sounds was operator-dependent and may be affected by background noise, distance from the doppler machine speaker, and static. Secondly, unlike with compressible vessels, there is no well-defined way to know the “ground truth” ABI value in non-compressible vessels. While our inter-rater reliability analysis of the 10 non-compressible vessel cases demonstrated substantial to excellent agreement for phasicity classification based on ABI machine waveform review (κ = 0.85, 95% CI: 0.51–1.00), with perfect concordance for monophasic and triphasic waveforms, the inherent subjectivity in waveform interpretation—particularly at the biphasic-triphasic interface—represents a limitation of using phasicity as ground truth. Nonetheless, waveform phasicity is a clinically reliable physiologic phenomenon and correlation with other metrics such as the TBI and PAT could be used as a surrogate measure of disease in validating assessment of calcified tibial arteries. The small sample size of non-compressible vessels (*n* = 10) limits the precision of our inter-rater reliability estimates, as reflected in the wide confidence interval, and a larger validation cohort is needed to more robustly establish the reliability of phasicity assessment in this challenging population. Thirdly, we did not integrate the demographics, such as patient medical history and prior interventions (presence of proximal bypass or stents) to account for differences in the hemodynamics, ABI values, or waveforms). Inclusion of baseline characteristics in future models would allow for a more sophisticated vascular disease prediction system.

We have demonstrated the feasibility of a novel deep learning-based approach for accurate prediction of ABI ranges through analysis of arterial sounds derived from hand-held dopplers. Significant validation work outside of the vascular lab in the “wild” at our center as well as other centers with a greater variety of continuous wave doppler probes is required. Once validated on a larger cohort of patients, this model could provide a low cost, objective, point-of-care solution to assess limb perfusion throughout the point-of-care continuum.

## Methods

### Clinical data collection

This prospective, IRB-approved study conducted at Stanford University involved the collection of continuous wave (CW) Doppler audio recordings paired with corresponding ABI values during standard noninvasive vascular assessments. Patients undergoing formal ABI testing provided informed consent and recordings were collected by registered vascular technologists (RVTs) using the Parks Flo-Lab 2100 Doppler system. The study cohort included patients referred for peripheral arterial disease (PAD) evaluation, encompassing both compressible and non-compressible vessels and including individuals with diabetes mellitus and other comorbidities affecting limb perfusion.

A custom-built iOS smartphone application deployed on iPhones was used to standardize Doppler sound recording during examinations. The built-in microphone was positioned directly adjacent to the Doppler probe. The app recorded audio at a sampling rate of 16,000 Hz in 4-second segments. It also included a logging interface for RVTs to annotate the audio clips with ground-truth ABI ranges (from the Parks Flo-Lab), waveform phasicity classification (monophasic, biphasic, or triphasic), laterality, artery type (posterior tibial or dorsalis pedis), and non-compressibility status. Non-compressibility was defined as ankle pressures exceeding 220 mmHg or surpassing the highest arm pressure by at least 40 mmHg.

All audio recordings from the study were screened for signal fidelity. Recordings with excessive ambient noise, clipped amplitudes, or indiscernible waveform patterns were excluded. All labels were verified by a board-certified vascular physician through a joint interpretation of the audio and visual spectrograms. Although patient-level demographic data were not collected, the dataset represented real-world heterogeneity across a vascular lab population. No specific patients were selected; instead, the unifying cohort characteristic was that all individuals presenting to the vascular lab with formal referrals for Doppler-based ABI examination during the study period were eligible for inclusion.

### Inter-rater reliability assessment for non-compressible vessels

Given the absence of reliable ABI measurements in non-compressible vessels, ABI testing waveform phasicity served as the ground truth reference for this subset. For all 10 non-compressible tibial artery cases, the corresponding ABI testing waveform recordings were independently evaluated by two board-certified vascular surgeons blinded to clinical outcomes and demographics. Each recording was classified as monophasic, biphasic, or triphasic based on recorded ABI testing waveform review. Inter-rater agreement was quantified using Cohen’s kappa coefficient. Cases with discordant classifications were adjudicated through consensus discussion.

### Phase detection and audio clip extraction

Each 4-second Doppler sound file was converted from stereo to mono and resampled to a uniform 16 kHz sampling rate. Amplitude normalization through log-transformation (in the range of 0 to 1) was applied to ensure consistent dynamic range across recordings. Peak detection was then performed using a moving-window strategy to locate systolic pulse peaks. Peaks were required to exceed a normalized amplitude threshold of 0.3 and be spaced by at least 1000 milliseconds to correspond to discrete cardiac cycles.

Each detected peak served as the temporal center for a 1-second audio clip, resulting in a series of phase-centered waveform segments per recording. These clips formed the standardized input units for downstream processing. Segmenting each 4-second file into multiple waveform phases substantially increased the number of training examples in the dataset while maintaining physiological relevance. Audio samples lacking detectable peaks were excluded.

### Sound processing and spectrogram conversion

**Fig. 4:**
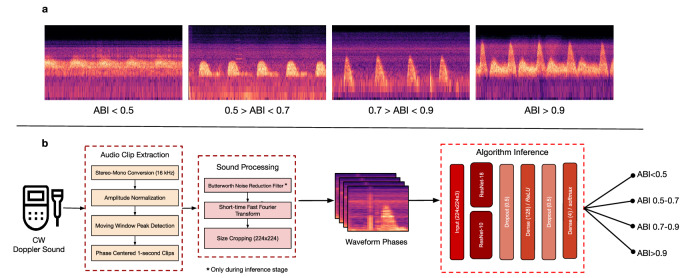
AutoABI system architecture. **a** Spectrograms representing temporal patterns that distinctly represent clinically significant ABI ranges, **b** Signal-processing-led deep learning pipeline for training and analysis of doppler sounds to predict ABI ranges.

Waveform segments were converted into mel-spectrogram images to facilitate classification using image-based deep learning architectures. The conversion of audio to spectrograms revealed temporal amplitude differentiation between the ABI ranges as seen in Fig. 4a. Short-time Fourier transform (STFT) was performed using a Hann window, a 25 millisecond frame length, and a 10 millisecond frame step. The resulting power spectrograms were projected onto 128 mel frequency bands with a maximum frequency of 8000 Hz. Following STFT computation, spectrogram power values were transformed to a logarithmic decibel (dB) scale using the maximum spectral power of each clip as the reference. Each spectrogram was then normalized to the range [0, 1] using min–max scaling on a per-clip basis to standardize dynamic range across samples while preserving relative intensity patterns

Each spectrogram was resized to 224 × 224 pixels and converted to an RGB image. This resolution was selected to retain visually distinct phase patterns while remaining computationally efficient for training. The model was trained directly on real-world recordings with variable noise with the goal of helping it generalize across naturally occurring diversity of clinical acoustic environments. A Butterworth high-pass filter (order 4, cutoff 30 Hz, sampling rate 16,000 Hz) was applied only during inference to reduce ambient low-frequency noise without removing temporal features that distinctly represent the ABI ranges.

### Deep learning and training procedure

To classify spectrograms into ABI categories, we implemented convolutional neural network models, as illustrated in Fig. 4b. All models accepted 224 × 224 × 3 spectrogram images as input. Two residual network architectures, ResNet-10 and ResNet-18, were constructed without pre-trained weights^16^. Feature representations extracted by the backbone network were passed through dropout regularization (rate 0.5), followed by a fully connected dense layer of 128 ReLU units, a second dropout layer (rate 0.5), and a final softmax classification layer producing probabilities over four ABI categories: <0.5, 0.5–0.7, 0.7–0.9, and >0.9.

Models were trained using a sparse categorical cross-entropy loss function and the Adam optimizer with a learning rate of 0.0001. The models were trained across 300 epochs with a batch size of 16. All experiments were conducted using an NVIDIA A100 graphics processing unit (GPU).

### Cross-validation and final model training

To evaluate model performance and generalization, we employed five-fold cross-validation. The dataset was randomly partitioned into five mutually exclusive subsets (folds), with each fold used exactly once as the held-out test set while the remaining four were used for training. This process resulted in five independently trained models, each evaluated on unseen data. One out of the five buckets was used for testing while the remaining four were used for training. Following cross-validation, a final model was trained on the entirety of the dataset (all five folds combined) using the same training hyperparameters and architecture as the cross-validation models. This test-folded cross-validation approach is outlined by Bradshaw et al.^17^ The final model, trained on the full dataset, was designated for deployment and downstream inference to maximize exposure to the complete training dataset. To prevent leakage of phases from the same patient being shared across training and test sets, cross-validation splits were constructed at the patient level, ensuring that all clips derived from a single patient were assigned exclusively to one fold. Consequently, each held-out test set (20% of patients per fold) contained only patients unseen during training.

### Model evaluation and statistical aggregation

Performance metrics were calculated for each fold independently and then aggregated. These included confusion matrices, per-class precision, recall, F1-scores, area under the receiver operating characteristic curve (AUROC), and testing accuracy. ROC curves were calculated using a one-vs-all strategy for each ABI category. Confusion matrices were normalized by row and expressed as percentages, and ROC curves were interpolated at 100 uniformly spaced false positive rate values. The 95% confidence intervals were generated using 1000 sample bootstrap resamples of each metric across all testing set predictions in the five-fold cross-validation.

## Supplementary information

Supplemental Materials

## Data Availability

The dataset collected from this study is not publicly available due to restrictions imposed by the IRB.
