# Supplementary material for: Enabling ankle-brachial index prediction from doppler sounds using deep learning
Source: NPJ Cardiovasc Health. 2026 Apr 9;3:21. doi: 10.1038/s44325-026-00116-7 (PMC13066085; doi:10.1038/s44325-026-00116-7)
Supplement: Supplementary file 1 — Supplemental Materials [file 44325_2026_116_MOESM1_ESM.pdf]

SUPPLEMENTAL MATERIAL:

Supplementary Table 1: Comparative Analysis of AutoABI versus Commercial Automated ABI Measurement Systems

Feature	AutoABI (Deep Learning-Based)	Commercial Automated Systems (e.g., MESI ABPI MD, Fukuda Form 5)
**Measurement Time**	30-60 seconds per limb (single doppler recording)	5-10 minutes per patient (4-limb automated cuff inflation)
**Setup Time**	Minimal (<1 minute)	Moderate (2-3 minutes for cuff placement and positioning)
**Total Clinical Time**	~2-5 minutes	~7-13 minutes
**Equipment Required**	Handheld CW Doppler ($200-500)<br>Smartphone with app (free)	Dedicated automated device ($3,000-15,000)<br>Multiple blood pressure cuffs<br>Integrated doppler system
**Equipment Portability**	Highly portable (pocket-sized doppler)	Semi-portable (wheeled cart or portable unit, 5-15 lbs)
**Operator Training**	Minimal (basic doppler technique)<br>No blood pressure measurement needed	Moderate (proper cuff sizing and placement)<br>Device-specific training

**Operator Burden**	Low (hold doppler over artery, record 4 seconds)	Low-Moderate (cuff placement, patient positioning, troubleshooting)
**Technical Skill Required**	Basic arterial localization with doppler	Proper cuff sizing and placement<br><br>Patient positioning
**Output Precision**	ABI range categories (<0.5, 0.5-0.7, 0.7-0.9, >0.9)	Precise ABI values (e.g., 0.87)
**Clinical Adequacy**	Sufficient for most triage and treatment decisions	Ideal for precise documentation and longitudinal monitoring
**Performance in Normal Vessels**	High discriminatory performance (average AUC: **0.97** for ABI > 0.9)	High accuracy (>95% correlation with manual ABI)
**Performance in PAD**	High discriminatory performance (average AUC: **0.94–0.96** across PAD ranges: <0.5, 0.5–0.7, and 0.7–0.9)	High accuracy in compressible vessels
**Calcified Vessels (>220 mmHg)**	Functional (predicts range based on waveform morphology)<br><br>100% concordance in pilot study (n=10)	Non-functional (falsely elevated ABI >1.4)<br><br>Requires alternative testing (TBI, PAT)
**Diabetes/ESRD Patients**	Potentially functional (requires validation)	Often fails due to medial arterial calcification

**Failure Mode - Calcification**	Continues to provide prediction based on acoustic features	Falsely elevated readings or "non-compressible" flag without alternative measurement
**Failure Mode - Arrhythmia**	May affect waveform quality (requires validation)	May affect oscillometric detection and accuracy
**Failure Mode - Obesity**	Minimal impact (doppler signal unaffected)	Difficult cuff sizing, reduced accuracy
**Failure Mode - Edema**	Minimal impact	May compress underlying tissues, affect accuracy
**Failure Mode - Ambient Noise**	Potential limitation (acoustic interference)	Minimal impact (pressure-based measurement)
**Integration into Workflow**	Point-of-care (bedside, clinic, ED)<br><br>Immediate triage	Typically vascular lab or dedicated room<br><br>Scheduled appointments
**Clinical Setting Suitability**	Emergency department<br><br>Outpatient clinic<br><br>Bedside<br><br>Home health<br><br>Resource-limited settings	Vascular laboratory<br><br>Primary care clinic with dedicated space<br><br>Well-resourced settings
**Reimbursement (US)**	Not yet established	Established (CPT 93922-93924)

**FDA Status**	Not yet submitted (investigational)	FDA-cleared Class II medical devices
**Capital Cost**	~$200-500 (if doppler not already owned)	$3,000-15,000
**Per-Test Cost**	Minimal (negligible computational cost)	Minimal (disposable supplies)
**Validation Status**	Single-center study (n=198 patients, 791 recordings)<br><br>Requires multi-center validation	Extensively validated across multiple centers and populations
**Primary Advantage**	Works in non-compressible vessels<br><br>Low cost<br><br>High portability<br><br>Rapid point-of-care assessment	Precise ABI values<br><br>FDA-cleared<br><br>Established reimbursement<br><br>Minimal operator dependence
**Primary Limitation**	Range categories (not precise values)<br><br>Requires validation across devices<br><br>Not yet FDA-cleared<br><br>Noise susceptibility	Fails in calcified vessels<br><br>Higher equipment cost<br><br>Less portable<br><br>Requires dedicated space

**Complementary Use Case**	Initial screening/triage<br><br>Non-compressible vessel assessment<br><br>Resource-limited settings<br><br>Emergency assessment	Definitive diagnosis<br><br>Precise longitudinal monitoring<br><br>Pre-procedural planning<br><br>Compressible vessels

Summary: AutoABI and commercial automated ABI systems represent complementary approaches with distinct advantages in different clinical contexts. Commercial systems provide precise ABI measurements in patients with compressible vessels and benefit from FDA clearance and established reimbursement pathways. AutoABI offers a novel solution for the clinically challenging population with calcified, non-compressible vessels where traditional pressure-based measurements fail, while also providing rapid point-of-care assessment in resource-limited settings. The ideal clinical implementation may involve using AutoABI for initial triage and non-compressible vessel assessment, with commercial systems reserved for precise measurement in compressible vessels and longitudinal monitoring.

Supplementary Table 2:

Inter-Rater Agreement for Phasicity Classification of Non-Compressible Tibial Artery Recordings

Recording Sample	Rater 1 Classification	Rater 2 Classification	Consensus Classification	Agreement
1	Monophasic	Monophasic	Monophasic	Yes
2	Monophasic	Monophasic	Monophasic	Yes
3	Monophasic	Monophasic	Monophasic	Yes
4	Monophasic	Monophasic	Monophasic	Yes

5	Biphasic	Triphasic	Biphasic	No
6	Biphasic	Biphasic	Biphasic	Yes
7	Triphasic	Triphasic	Triphasic	Yes
8	Triphasic	Triphasic	Triphasic	Yes
9	Triphasic	Triphasic	Triphasic	Yes
10	Triphasic	Triphasic	Triphasic	Yes

Notes: Recording sample 5 showed discordance where Rater 1 classified the waveform as biphasic while Rater 2 classified it as triphasic. Consensus review confirmed biphasic classification based on subtle dampening of the reverse flow component on the ABI machine waveform tracing.
